# Effects of Tea Plant Varieties with High- and Low-Nutrient Efficiency on Nutrients in Degraded Soil

**DOI:** 10.3390/plants12040905

**Published:** 2023-02-16

**Authors:** Li Ruan, Xin Li, Yuhang Song, Jianwu Li, Kumuduni Niroshika Palansooriya

**Affiliations:** 1 Institute of Sericulture and Tea, Zhejiang Academy of Agricultural Sciences, Hangzhou 310021, China; 2 Institute of Carbon Neutrality, Zhejiang A&F University, Hangzhou 311300, China; 3 Agricultural Technology Extension Station of Tangshan Agricultural and Rural Bureau, Tangshang 063000, China; 4 State Key Laboratory of Subtropical Silviculture, Zhejiang A&F University, Hangzhou 311300, China

**Keywords:** tea plant varieties, soil nutrient, soil degradation, soil profile

## Abstract

Tea plants are widely planted in tropical and subtropical regions globally, especially in southern China. The high leaching and strong soil acidity in these areas, in addition to human factors (e.g., tea picking and inappropriate fertilization methods) aggravate the lack of nutrients in tea garden soil. Therefore, improving degraded tea-growing soil is urgently required. Although the influence of biological factors (e.g., tea plant variety) on soil nutrients has been explored in the existing literature, there are few studies on the inhibition of soil nutrient degradation using different tea plant varieties. In this study, two tea plant varieties with different nutrient efficiencies (high-nutrient-efficiency variety: Longjing43 (LJ43); low-nutrient-efficiency variety: Liyou002 (LY002)) were studied. Under a one-side fertilization mode of two rows and two plants, the tea plant growth status, soil pH, and available nutrients in the soil profiles were analyzed, aiming to reveal the improvement of degraded soil using different tea varieties. The results showed that (1) differences in the phenotypic features of growth (such as dry tea yield, chlorophyll, leaf nitrogen (N), phosphorus (P), and potassium (K) content) between the fertilization belts in LJ43 (LJ43-near and LJ43-far) were lower than those in LY002. (2) RDA results showed that the crucial soil nutrient factors which determine the features of tea plants included available P, slowly available K, and available K. Moreover, acidification was more serious near the fertilization belt. The pH of the soil near LJ43 was higher than that near LY002, indicating an improvement in soil acidification. (3) Soil nutrient heterogeneity between fertilization belts in LJ43 (LJ43-near and LJ43-far) was lower than in LY002. In conclusion, the long-term one-side fertilization mode of two rows and two plants usually causes spatial heterogeneities in soil nutrients and aggravates soil acidification. However, LJ43 can reduce the nutrient heterogeneities and soil acidification, which is probably due to the preferential development of secondary roots. These results are helpful in understanding the influence of tea plant variety on improving soil nutrients and provide a relevant scientific reference for breeding high-quality tea varieties, improving the state of degraded soil and maintaining soil health.

## 1. Introduction

The tea plant (*Camellia sinensis* (L.)) is a perennial evergreen economic forest crop that is widely planted in tropical and subtropical regions of the world, especially in the south of China [1,2,3]. The soil in these areas is generally highly leachable and strongly acidic, resulting in a lack of nutrients in tea garden soil [4]. The lack of soil nutrients in tea gardens has been further exacerbated by human factors, such as the large amount of nutrients taken away by tea picking and inappropriate fertilization methods; these human factors have become universally limiting for tea production [5]. In recent years, the influence of biological factors (e.g., plant variety) on soil nutrients has been explored by the existing literature [6,7]. In terms of the anthropogenic influence, cultivation methods (including selection of cultivars) have a key impact on the ecological environment of the soil [8].

Since plants and soils have mutual influence and coordinate their development, variation in plant species and quantity can be triggered by different soils [9]. Likewise, soil development is affected by the cultivated plant species [10]. Variation in soil fertility, including the difference in root growth, litter accumulation, and animal and microorganism species, is closely related to the plant’s species. The root system, which functions as the link between plants and soil, influences the delivery of soil nutrients to the plants and the distribution of nutrients in the soil. In this regard, the variation of plant parameters is a crucial factor influencing the variation in soil fertility. Different kinds of plant roots have different effects on soil surface water, soil bulk density, and shear strength [11]. Moreover, there is a positive relationship between root biomass and soil nutrients, representing different influences of plant species on soil properties [12]. For some plants (such as legumes and sea buckthorn), the root system, with its unique nitrogen fixation function, helps to accelerate the cycling process of soil nutrients [13]. Furthermore, the variation in nutrient availability in soil is driven by the different physiological reactions in roots (such as differences in the type and content of organic acids and the activity of root microorganisms), which influence the distribution of nutrients in soil [14,15,16]. Therefore, it is obvious that plant species are crucial when it comes to stimulating the development and evolution of soil.

Even if there is a relatively smaller difference in plant genotypes compared with that of plant species, the influence of plants with different genotypes on soil features should not be neglected [17]. First, plants with different genotypes generate different influences on the distribution of soil nutrients due to the different abilities of root systems to absorb soil nutrients [18,19]. It has been revealed by previous research on *Glycine max*, *Arabidopsis*, *Oryza sativa*, and other plants that there are obvious differences in the distribution features of soil nutrients for plants of different genotypes due to differences in nutrient absorption abilities, such as root morphology, root exudates, root activity, and so on [20,21]. Second, the root exudates of different varieties of plants are significantly different, which leads to relatively larger differences for the microbial community in the rhizosphere soil of plants. This affects the organic matter content of soil, the soil nutrient cycle, and soil productivity (e.g., blueberries, corn, and rice) [21,22,23,24]. Thus, plant type can exert a crucial influence on the features of soil by transforming the rhizosphere environment by way of, for example, nutrient absorption, generation of debris, and root exudates [22].

The physical and chemical properties of the soil in tea gardens after undergoing years of plantation are gradually transformed, and the unique eco-environment in the soil will be generated accordingly thanks to the unique biological features of tea plants [25,26]. Current research on how to reduce soil nutrient degradation in tea gardens using different tea plant varieties is relatively lacking, but is very important to ensure the green, healthy, and sustainable development of tea plantations. Previous studies have reported that there are two tea plant genotypes (i.e., Longjing43 (LJ43) and Liyou002 (LY002)) which are endowed with different nutrient absorption abilities [25]. Based on these findings, it can be further speculated that LJ43 and LY002 will have different influences on the distribution of soil nutrients. Therefore, this study aims to (1) explore the growth phenotype and leaf nutrient content of the above two tea plant genotypes and the nutrient distribution characteristics in the soil profile under a single-side fertilization mode of two lines and two plants, and (2) reveal the influence of tea plants with different genotypes on soil nutrient distribution. These results are conducive to understanding the influence of tea plant varieties on improving soil nutrients and provide relevant scientific reference for breeding high-quality tea varieties, ensuring the green, healthy, and sustainable development of tea plants and the alleviation of soil degradation.

## 2. Results

### 2.1. The Phenotypic Features of Growth for the Two Tea Plants

**Figure 1:**
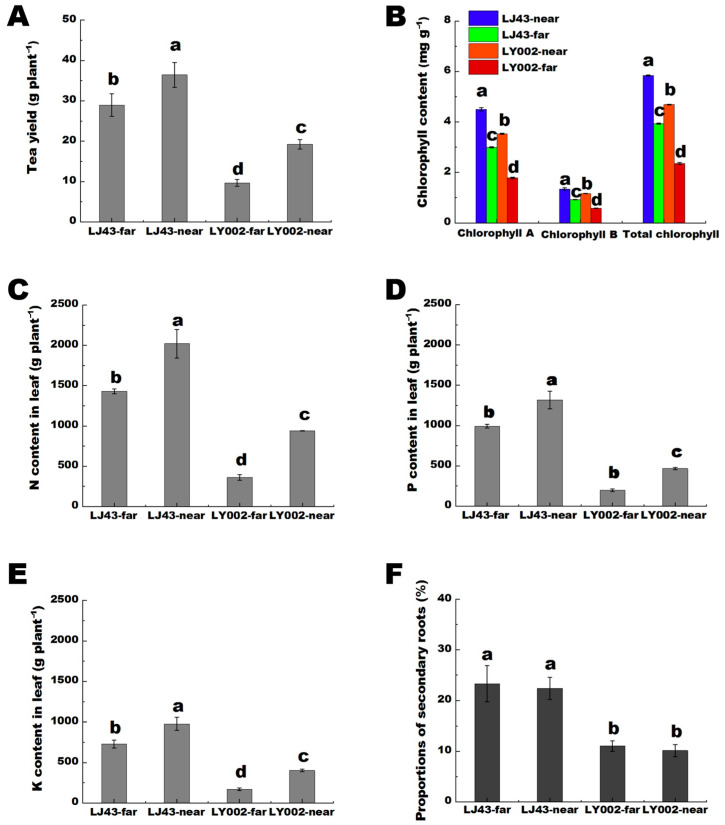
The differences in growth parameters of two tea genotypes under one-side fertilization mode of two rows and two plants. (**A**): dry tea yield; (**B**): chlorophyll content in leaves; (**C**): N content in leaves; (**D**): P content in leaves; (**E**): K content in leaves; (**F**): proportion of secondary roots. Data shows the mean ± standard deviation (*n* = 3); there was a significant difference, *p <* 0.05, where the mean value is represented by different letters.

The phenotypic features of growth for the two tea plants are shown in Supplementary Figure S1B. For LJ43 tea plants, the far (LJ43-far) and near (LJ43-near) fertilization belt had similar phenotypic features of growth, including leaf color and germination density, whereas the LY002, the LY002-far had shallower leaf color and lower germination density compared with that of the LY002-near. In terms of the yield of dry tea, LJ43-far was 80% of LJ43-near, whereas LY002-far was merely 50% of LY002-near (Figure 1A). In terms of chlorophyll A, chlorophyll B, and total chlorophyll content, LJ43-far was above 65% of LJ43-near, whereas LY002-far was merely 50% of LY002-near (Figure 1B). In terms of the contents of N, P, and K in leaves, LJ43-far was above 70% of LJ43-near, whereas LY002-far was merely 50% of LY002-near (Figure 1C–E). The proportion of secondary roots was significantly higher for LJ43 than for LY002 (*p* < 0.05) (Figure 1F).

### 2.2. Correlation between the Features of Soil Nutrient and Tea Plants

**Figure 2:**
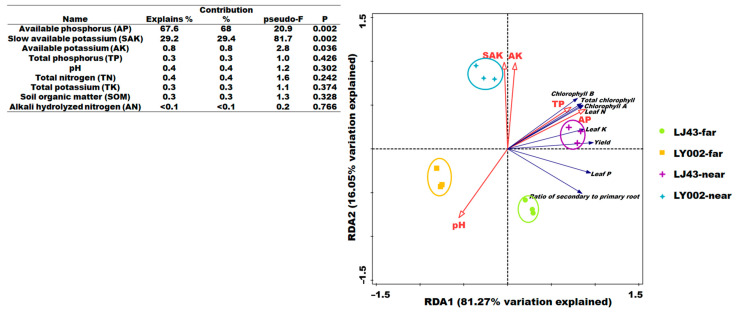
RDA ranking of soil nutrient and tea plant features.

The results of RDA analysis between the soil nutrient features and tea plants (LJ43 and LY002) are presented in Figure 2. Regarding the four data groups, LJ43-far, LY002-far, LJ43-near, and LY002-near were relatively separated between different groups but clustered within their respective groups. These results indicate that there was good data repeatability and obvious differences between the data in the four groups. There was a small difference between LJ43-far and LJ43-near but a large difference between LY002-far and LY002-near (Figure 2), further indicating that the difference in soil nutrients between the far and near fertilization belts was less for LJ43 than for LY002. From the vertical distance from the sampling point to the arrow of soil nutrient factor, it can be seen that the soil pH values were negatively correlated with the distance to the fertilization belts. However, other soil nutrient features (e.g., available P, slowly available K, available K, and total P) were positively correlated with the distance to the fertilization belts. The angle between the arrow of various tea plant features and the arrow of soil nutrient features showed that the soil nutrient factors (e.g., total P and available K) had a positive relationship with the features of tea plants (e.g., tea yield; Figure 2). There was a negative correlation between features of tea plants and pH value. The angle between the soil nutrient arrows showed a positive relationship between the other nutrition features, except for pH value. This indicated that the crucial soil nutrient factors determining the features of tea plants included available P, slowly available K, and available K (*p <* 0.05) under the long-term single-side fertilization mode. Accordingly, the features of variation in pH value, available P, available K, and slowly available K should be highlighted in soil profiles.

### 2.3. Distribution Features of Crucial Nutrients in Soil Profile

**Figure 3:**
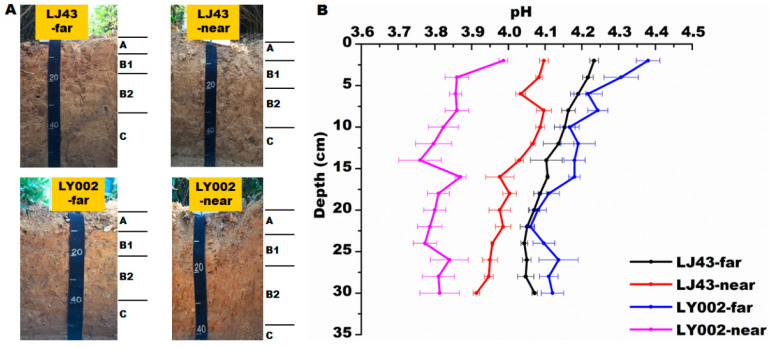
Soil profile and variation in pH of soil related to LJ43 and LY002 with different treatments. Data shows the mean ± standard deviation (*n* = 3). (**A**): soil profile for each treatment; (**B**): variation in pH in each treatment.

Before and after the test, the distribution of pH values in the soil profile for the growth of the two tea plants showed that there was a moderate decrease in soil pH, whereas the decrease in soil pH in the near fertilization belts (-near) was more severe (Figure 3). The soil pH in each layer decreased by 0.39–0.59 and 0.57–0.77, compared with pH levels before the test, for LJ43-near and LY002-near, respectively. Regarding near (former) and far (latter) from the fertilization belts, the soil pH of the two tea plants in the former was lower than in the latter. As for LJ43, the soil pH of far from and near to the fertilization belts was relatively close, and pH values in each layer of LJ43-near were decreased by 0.07–0.16 compared with those of LJ43-far. In the case of LY002, the difference in soil pH between the far and near fertilization belts was relatively larger, and soil pH values in each layer of LY002-near were reduced by 0.27–0.45. The pH values in each layer of LJ43-near were higher than those of LY002-near (Figure 3).

**Figure 4:**
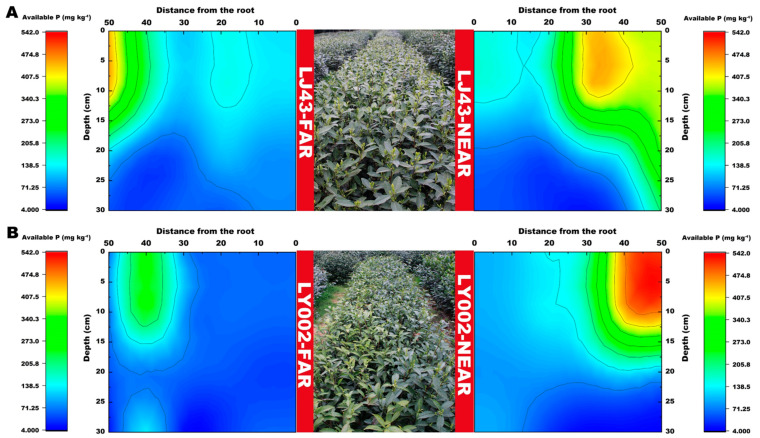
Variation in soil available P content related to LJ43 and LY002 with different treatments. (**A**): LJ43; (**B**): LY002. Data show the mean ± standard deviation (*n* = 3). The fertilization zone is located at the right-most side (i.e., 50 cm distance to LJ43-near or LY002-near main roots). Regarding the tea plants near the fertilization belts (LJ43-near or LY002-near), the fertilization belt was 50 cm distance from main roots of the tea plants; regarding the tea plants far from the fertilization belts (LJ43-far or LY002-far), the fertilization belt was 100 cm distance from the main roots of the tea plants.

The distribution features of available P in the soil profile for the two tea plants are presented in Figure 4. The relative enrichment area of available P (>400 mg kg^−1^) was 30–40 and 50 cm distance from the main roots of the tea plants for LJ43-near and LY002-near, respectively (Figure 4A,B). The relative enrichment area of available P (>400 mg kg^−1^) was 45–50 and over 50 cm distance from the main roots of the tea plants for LJ43-far and LY002-far, respectively.

**Figure 5:**
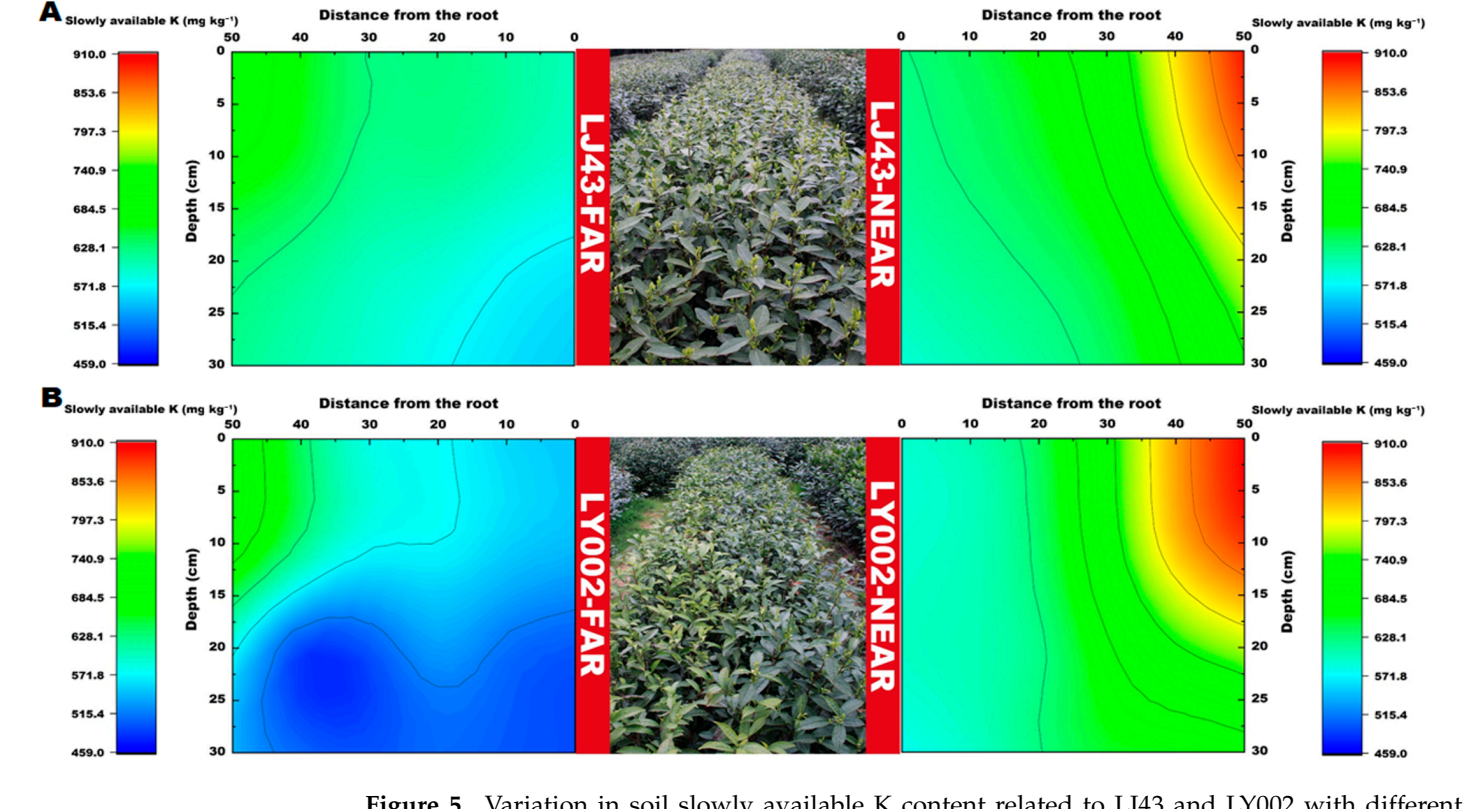
Variation in soil slowly available K content related to LJ43 and LY002 with different treatments. (**A**): LJ43; (**B**): LY002. Data shows the mean ± standard deviation (*n* = 3).

**Figure 6:**
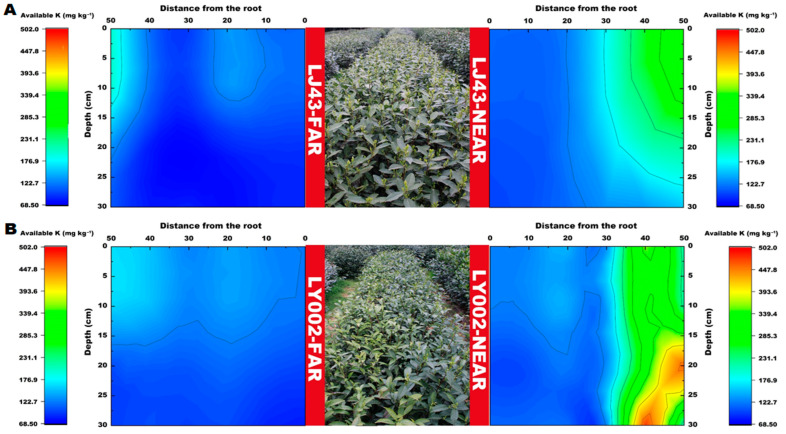
Variation in soil available K content related to LJ43 and LY002 with different treatments. (**A**): LJ43; (**B**): LY002. Data shows the mean ± standard deviation (*n* = 3).

The distribution features of slowly available K in the soil profile for the two tea plants are presented in Figure 5. The slowly available K content of the two tea plants near the fertilization belt (LJ43-near or LY002-near) gradually decreased from the 50 cm distance from the fertilization belt to the roots. Moreover, the relative enrichment areas of slowly available K (>850 mg kg^−1^) were all concentrated within 40–50 cm of the main roots of the tea plants. The slowly available K in the LJ43-near soil, at 0–10 cm distance to the main roots, had a relatively larger variation in the horizontal direction than in the vertical section. The slowly available K in the LJ43-near soil had a larger variation in the tillage layer (0–20 cm) than in the lower layer, whereas the slowly available K in the LY002-near soil had a small variation in the entire vertical section, which was smaller than that of LJ43-near. The relative enrichment area of slowly available K in the LJ43-far soil, which was far away from the fertilization belt (>620 mg kg^−1^), was relatively expansive, whereas the slowly available K in the LY002-far soil was relatively narrower compared with that in the enrichment area. The slowly available K in most LJ43-far-area soils, at 0–30 cm distance to the main roots in the horizontal direction, was above 600 mg kg^−1^, whereas the slowly available K in most LY002-far area soils was above 600 mg kg^−1^. In addition, the relative enrichment areas of available K (>200 mg kg^−1^) were concentrated 30–50 and 35–50 cm away from the main roots of the tea plants for LJ43-near and LY002-near, respectively (Figure 6A), and their content gradually changed along the distance from the fertilization belt for LJ43-near. The largest enrichment area of available K was 15–30 cm away from the ground (Figure 6B). The relative enrichment areas of available K were 45–50 cm away from the main roots for LJ43-far, and the content of available K was above 175 mg kg^−1^, whereas the available K was above 150 mg kg^−1^ for LY002-far.

### 2.4. Variation Coefficient of Soil Nutrient Indicators

The variation coefficients of the soil nutrients of LJ43 were less than those of LY002 in the far and near fertilization belts (Table 1). There was an obvious difference in the variation coefficients of soil pH, available P, available K, and total K between the far and near fertilization belt (*p* < 0.01). The variation coefficients of soil pH, available P, available K, and total K between the far and near fertilization belts of LY002 were 1.5, 1.4, 1.7 and 1.6 times higher than those of LJ43, respectively. It was revealed that LJ43 plantation was able to relatively reduce the differences of soil nutrients in the far and near fertilization belts.

## 3. Discussion

The long-term one-side fertilization mode of two rows and two plants was able to lead to the differentiation in the physical and chemical properties of the soil in the far and near fertilization belts. Significantly, the effects of fertilization zone distance on different varieties of tea plants were obvious. For the high nutrient efficiency genotype (LJ43), the distance to the fertilization belt had little influence on their growth status in fields, yield of dry tea, leaf chlorophyll, N, P, and K contents of the two tea plants, whereas distance had a greater influence on the low nutrient efficiency genotype (LY002). The ratio of secondary and primary roots of LJ43-far and LJ43-near was obviously higher than that of LY002-far and LY002-near. In addition, the development of secondary roots can help determine the ability of tea plants to nutrient-capture [27]. Secondary rooting can promote plants to absorb soil nutrients because they have a large root surface area, which helps to increase the contact area between roots and soil [28,29]. In this study, LJ43 developed secondary roots earlier than LY002 far and near the fertilization belt. Furthermore, the contents of N, P, and K in the leaves of LJ43 were obviously higher than in the leaves of LY002. Ruan et al. [25] indicated that LJ43 had a higher ability in efficiency of nutrient absorption than that of LY002, which was possibly related to its feature of excelling in developing secondary roots. Obviously, the prioritized development of secondary roots in LJ43 was conducive to promoting its absorption of soil nutrients. On the other hand, with the development of secondary roots, LJ43 was also conducive to enhancing the distribution of the roots of tea plants in the soil, and was therefore able to promote tea plants to absorb more nutrients in the soil as well as speed up the reorganization of soil nutrients in the soil profile [28,29]. The RDA analysis results showed that the difference in soil nutrients in the far and near fertilization belt was LJ43 > LY002 (Figure 2). Meanwhile, the variation coefficient of soil nutrients showed that the variation coefficient of soil nutrients of LJ43 in the far and near fertilization belt was higher than that of LY002 (Table 3). Apparently, LJ43 was able to decrease the differences in soil nutrient features in the far and near fertilization belt, with the ability possibly originating from the prioritized development of secondary roots.

Secondly, the pH and bulk density indicated soil degradation in the tea garden. Compared with CK, the pH decreased by 0.45–0.79 units, while the bulk density increased by 0.03–0.26 g cm^−3^. Compared with CK, the total P, total K, and CEC decreased by 0.2–0.56 g kg^−1^, 0.2–0.62 g kg^−1^, and 0.7–1.1 (cmol(+) kg^−1^, respectively (Table 2). This indicated that the tea garden soil was degraded. The degree of soil acidification caused by tea gardens and fertilization will vary with different tea plant varieties. RDA analysis showed that the other nutrient features had a positive relationship except for soil pH, which is a crucial and basic property of soil, as well as one of the factors affecting soil fertility (Figure 2). The topdressing urea application promoted the development of soil acidification over the long term [30], and ammonium and H^+^ release from tea plants led to the intensification of soil acidification. There was a reduction in the mean value of soil pH for tea plants to some extent before and after the experiment compared with the mean value calculated before this study was conducted. The varieties of tea plants, rather than distance of fertilization zone, had greater effect on soil pH value (Figure 3).

In addition, different tea varieties had an important impact on the distribution of crucial soil nutrient factors. The crucial soil nutrient factors that determine the characteristics of the tea plants in this study included available P, slow available K, and available K, rather than alkali hydrolysable N and total N, for the following reasons: (1) tea plants were picked and pruned every year to remove a large amount of nitrogen [31]; (2) the base and top fertilizers used in this study were rapeseed cake (N ≥ 5.25%, P_2_O_5_ ≥ 3.91%, K_2_O ≥ 2.7%) and urea (N 46%), and the annual total nitrogen input is much higher than that of P and K [32]; and (3) the order of soil nutrient mobility was ranked as N > K > P, and N move rapidly and was absorbed by tea plant roots accordingly, whereas P and K are easily fixed and are hardly absorbed by tea plant roots in this regard [33]. Thus, the crucial soil nutrient factors that determined the differences in tea plant features in the research were P and K, as opposed to N. As K and P move slowly in the soil, their availability and distribution range in the soil is greatly influenced by root system morphology [34]. In the case of P, the excretion of P mobilizing agents may be relevant considering the cultivar-dependent P effect on plant yield [14]. For the varieties with high nutrient efficiency, soil nutrients can be effectively absorbed by maintaining good root system morphology [35]. In this study, LJ43 performed better in developing secondary roots, which have relatively greater advantages than primary roots in terms of nutrient absorption. For instance, secondary roots have stronger root vitality and larger absorption areas [36], so it is possible for them to effectively enhance the nutrient absorption efficiency of plants. It was revealed by the results obtained in both this study, as well as previous studies, that LJ43 has a higher nutrient absorption efficiency than that of LY002. The contents of available P and slowly available K in the LJ43 soil near to and far from the fertilization belts, within 0–10 cm of the root system in horizontal direction (LJ43-near and LJ43-far), were higher than those for LY002 (Figure 4 and Figure 5). Furthermore, the available P and slowly available K in the LJ43 soil were relatively concentrated within the topsoil layer (0–15 cm). From the perspective of the spatial distribution of available K, the available K in the LY002 soil near the fertilization belt was concentrated blow 15 cm (Figure 6). It seems that LJ43 has a better ability to lead P and K from the fertilization belt to the root system, so P and K were relatively concentrated near the root system of LJ43. This may be related to the relatively denser secondary roots of LJ43 being more evenly distributed in the soil, which makes it possible for LJ43 to absorb more P and K from the soil. There was a concentration gradient of P and K being formed in the soil far from and near to the root systems, which promoted the diffusion and mobility of P and K in the soil.

## 4. Materials and Methods

### 4.1. Plant and Soil Materials

Field trials were carried out in a place close to Yunqi Zhujing in Hangzhou, Zhejiang Province, Southeast China, which is a major production area for class-I Longjing tea in China [37]. The area is categorized as having a subtropical monsoon climate with an annual average precipitation of 1139 mm and an annual average temperature of 17.5 °C [38]. The soil parent material is river alluvium, and the area has flat ground and valley terraces, with an altitude of about 30 m and a slope of about 1.5°. The soils are classified as Luvisols according to Chinese Soil Taxonomy [39]. The tested soil background is comparable according to the soil profile morphology, which provides a prerequisite for the subsequent comparative study of different treatments. Before the test, the basic physical and chemical properties in 0–30 cm of soil were determined, as follows: pH 4.4–4.6, bulk density 1.18–1.29 g cm^−3^. According to the United Nations soil texture classification, the particle size distribution was as follows: clay (<2 μm) 18.7–20.8%, silt (2–63 μm) 73.5–75.7%, sand (>63 μm), 4.6–5.2%.

**Figure 7:**
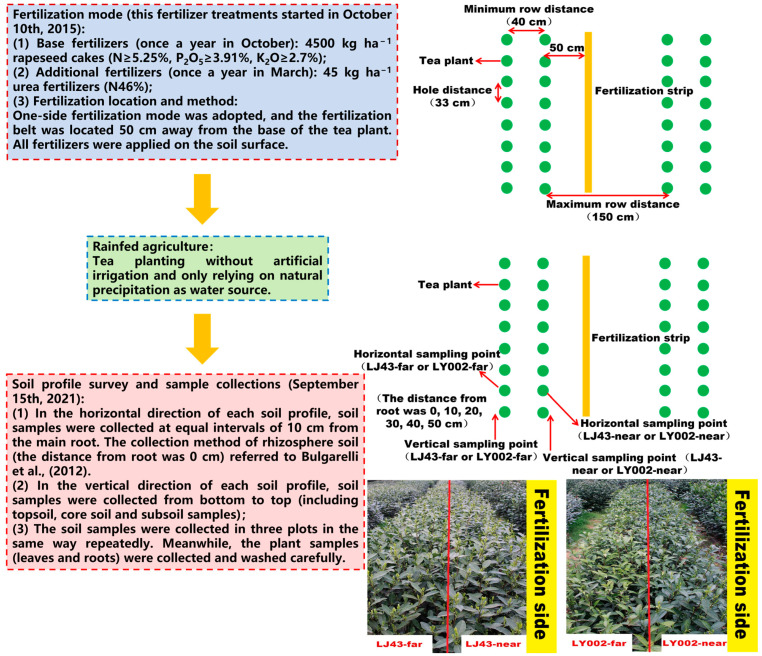
The flowchart of the whole experiment (including when applied treatments, irrigation, fertilization, when sampling of soil or plants and how, etc.) [25,41].

For the purposes of this study, two tea varieties (Longjing43 (LJ43) and Liyou002 (LY002)) were offered by the Tea Research Institute, Chinese Academy of Agricultural Sciences. According to previous studies [25], these two tea genotypes have different nutrient efficiencies (high-nutrient-efficiency variety: Longjing43 (LJ43); low-nutrient-efficiency variety: Liyou002 (LY002)). A previous study [25] described the tea planting mode and fertilization treatment in detail (Figure 7): 14-month-old tea cuttings were planted in unilaterally fertilized double rows in the experimental tea garden (i.e., with the cultivation of double plants at equal distances; the minimum row distance was 40 cm, the maximum row distance was 150 cm, the hole distance was 33 cm, and the fertilizer was side-dressed on the unilateral root base) (Supplementary Figure S1A). In October of every year of the study period, 4500 kg ha^−1^ rapeseed cakes (N ≥ 5.25%, P_2_O_5_ ≥ 3.91%, K_2_O ≥ 2.7%) were used as basic fertilizers. In March of every year of the study period, 45 kg ha^−1^ urea fertilizer (N46%) was used as additional fertilizer. The above fertilizer treatments started on 10 October 2015. The tea plants close to the fertilization belt were marked as -near in accordance with its distance to the fertilization belt; the tea plants far from the fertilization belt were marked, accordingly, as -far. The soil field investigation and multiple cross-sectional interviews were carried out on 15 September 2021. According to the field investigation, four typical soil profiles were selected: LJ43-near, LJ43-far, LY002-near, and LY002-far, representing the near and far fertilization belt for LJ43 and LY002, respectively. In the horizontal direction of each soil profile, soil samples were collected at equal intervals of 10 cm from the main root. In the vertical direction of each soil profile, soil samples were collected from bottom to top (including topsoil, core soil, and subsoil samples). The soil samples were collected from three plots in the same way, repeatedly. Meanwhile, the plant samples (leaves and roots) were collected and washed carefully. The soil profiles and layer divisions are shown in Figure 3A. The description for the root collections was as follows: Firstly, the representative tea plants were selected. The whole root systems were carefully excavated from the soil. The root systems were separated from the aboveground parts. The attached soil on the roots was carefully shaken off, and the rhizosphere soils were brushed down. Secondly, the root systems were taken back to the laboratory for cleaning and the primary and secondary roots were carefully separated. Thirdly, the cleaned primary and secondary roots were dried at 80 °C for 30 min and dried at 65 °C for 24 h. Finally, the primary and secondary roots were pulverized and weighed. The description and characterization of the soil profiles are shown in Table 3 and Figure 3A. The specific method of soil profile sampling has been described in previous studies [40].

### 4.2. Treatments of Samples and Analysis in Lab

To determine the tea yield, young shoots with one bud and two leaves were collected from each tea row during the spring tea period; they were spread and exposed overnight, then underwent tea green removal at 220–280 °C using a hand rolling process in a Longjing pot. They were baked for 5 min at 120 °C with a 6CHM-901 electric heating dryer, then dried at 80 °C to constant weight. Finally, they became the dried green tea sample for weighing the dry tea output. The dry tea yield per plant was calculated in accordance with the total tea plants in the tea row.

Thirty mature leaves were selected and measured using the acetone method [42]. Then, 0.100 g of fresh leaf sample was added into 10 mL of extraction solution (V acetone:V95% ethanol = 1:1) and the liquid was shaken evenly with the soaking extract, in the dark, at room temperature, for 24 h. After that, it was centrifuged at a speed of 3000 r min^−1^ for 10 min. A SHIMADZU UV-2550 spectrophotometer was used to measure the light absorption values at 663 nm and 645 nm. Measurements were repeated three times for each sample. The chlorophyll content was measured using the modified formula of the Arnon method [35]:

(1) $$Ca=(12.71D_{663}-2.59D_{645})V/m$$

(2) $$Cb=(22.88D_{645}-4.67D_{663})V/m$$

(3) $$Ct=(8.04D_{663}+20.29D_{645})V/m$$

*C*a is the content of chlorophyll a (mg g^−1^), *C*b is the content of chlorophyll b (mg g^−1^), and *C*t is the total amount of chlorophyll (mg g^−1^). *D*_645_ and *D*_663_ represent the optical density at the wavelengths of 645 nm and 663 nm, respectively, *V* is the constant volume (mL), and *m* is the sample weight (g).

To determine the nutrient content in leaves, the cleaned leaves were initially dried at 80 °C for 30 min, and subsequently dried at 65 °C for 24 h. Then, the leaves were pulverized. The crushed leaves were digested using the H_2_SO_4_-H_2_O_2_ digestion method. After that, the Kjeldahl method, vanadium molybdenum yellow colorimetry, and flame photometry were used to measure the N, P, and K of the leaves [43,44].

Plant roots and litter were picked out from the collected soil samples after they had dried naturally. Then, soil samples were taken using the quartering method and recollected for further use after being ground and filtered through 10 mesh-, 60 mesh-, and 100 mesh-nylon screens, in that order. The physical and chemical properties of the soil (e.g., pH value, soil organic matter, alkali hydrolyzed N, available P, available K, slowly available K, and total N, P, and K) were measured according to the methods outlined by Zhang and Gong [27].

### 4.3. Statistical Analysis

For statistical analysis, SPSS 18 and Microsoft Excel 2019 were employed. Origin 9.0 was used for drawing. One-way ANOVA was used to analyze the significance of differences in the data. Redundancy analysis (RDA) between soil nutrients and tea plant features was performed using Canoco 5.0 (Microcomputer Power, Clover Lane, Ithaca, NY, USA).

## 5. Conclusions

The high leaching and strongly acidic soils in tropical and subtropical regions, tea picking methods, and inappropriate fertilization methods, aggravate the lack of nutrients in tea garden soil. Through a high-nutrient-efficiency variety of tea plant (LJ43), both nutrient heterogeneity and soil acidification can be reduced, so as to greatly improve the soil degradation in tea gardens. The results show that the preferential development of secondary roots makes LJ43 efficiently absorb nutrients in the soil, promoting nutrient diffusion and migration in the soil, providing a chance for the plant to adapt to the nutrient spatial heterogeneity, and promoting efficient rapid nutrient absorption. Furthermore, long term emphasis on N fertilizer aggravates soil acidification, and so P and K become the crucial nutrient factors in soils that determine the tea plant features. LJ43 performs excellently in alleviating such soil acidification. This study lays a theoretical foundation for understanding the efficient utilization of soil nutrients by tea plant varieties. It also provides a relevant reference for improving soil nutrients using tea plant varieties, for breeding high-quality tea varieties, and for improving the state of degraded soil.

## Figures and Tables

**Table 1:** Variation coefficients of various soil nutrient indicators.

Variety	pH	SOM	AP	AK	AN	SAK	TN	TP	TK
LJ43	6.3 ± 0.1 **	25.3 ± 1.9	94.2 ± 4.3 **	43.1 ± 0.4 **	48.5 ± 1.6	17.0 ± 0.1 **	39.5 ± 2.3 *	16.2 ± 0.6	30.1 ± 1.4 **
LY002	9.2 ± 0.2	31.0 ± 3.1	130.3 ± 4.0	73.9 ± 5.6	47.8 ± 2.4	16.2 ± 0.2	56.9 ± 3.5	17.7 ± 0.6	46.8 ± 1.6

Note: SOM, AP, AK, AN, SAK, TN, TP, and TK stand for soil organic matter, available phosphorus, available potassium, alkali hydrolyzable nitrogen, slowly available potassium, total nitrogen, total phosphorus and total potassium, respectively. ** and * indicate significant differences at *p <* 0.01 and *p <* 0.05 levels, respectively.

**Table 2:** Parameters indicating soil degradation.

Parameter	CK	LJ43-Far	LJ43-Near	LY002-Far	LY002-Near
pH	4.62 ± 0.05 a	4.11 ± 0.04 c	4.01 ± 0.04 d	4.17 ± 0.05 b	3.83 ± 0.03 e
Bulk density (g cm^−3^)	1.17 ± 0.06 b	1.20 ± 0.06 ab	1.28 ± 0.05 ab	1.27 ± 0.06 ab	1.36 ± 0.04 a
Total P (g kg^−1^)	2.18 ± 0.02 a	1.77 ± 0.01 c	1.98 ± 0.02 b	1.62 ± 0.01 c	1.88 ± 0.01 b
Total K (g kg^−1^)	5.28 ± 0.05 a	4.94 ± 0.03 c	5.08 ± 0.05 b	4.66 ± 0.02 d	5.05 ± 0.04 b
CEC (cmol(+) kg^−1^)	13.8 ± 0.06 a	13.1 ± 0.05 b	12.9 ± 0.02 c	13.0 ± 0.04 b	12.7 ± 0.01 d

Note: Total P and K represent the concentration of total P and K, respectively. CEC represents cation exchange capacity. CK represents the original soil without tea planting and fertilization. The data show the characteristics of the soil profile. Data are means ± SEs (n = 3). Different letters represent significant differences at *p* < 0.05 levels.

**Table 3:** Description of soil profile morphology in Yunqi Zhujing, Hangzhou, China.

Soil Profile	Horizon	Color	Structure	Soil New Growth	Root System
LJ43-far	A	7.5YR3/4	Granular	A small amount of iron-manganese spots	Many
	B1	10YR7/6	Blocky	A small amount of iron-manganese spots	Many
	B2	10YR5/6	Blocky	More iron-manganese spots and iron-manganese concretion	Common
	C	10YR5/6	Blocky	A small amount of iron-manganese spots	Few
LJ43-near	A	7.5YR3/4	Granular	A small amount of iron-manganese spots	Many
	B1	10YR7/6	Blocky	A small amount of iron-manganese spots	Many
	B2	10YR5/8	Blocky	More iron-manganese spots and iron-manganese concretion	Common
	C	10YR5/8	Blocky	A small amount of iron-manganese spots	Few
LY002-far	A	7.5YR3/4	Granular	A small amount of iron-manganese spots	Many
	B1	10YR6/6	Blocky	More iron-manganese spots and iron-manganese concretion	Many
	B2	10YR4/6	Blocky	More iron-manganese spots and iron-manganese concretion	Common
	C	10YR5/7	Blocky	A small amount of iron-manganese spots	Few
LY002-near	A	7.5YR3/4	Granular	A small amount of iron-manganese spots	Many
	B1	10YR6/6	Blocky	More iron-manganese spots and iron-manganese concretion	Many
	B2	10YR5/7	Blocky	More iron-manganese spots and iron-manganese concretion	Common
	C	10YR5/7	Blocky	A small amount of iron-manganese spots	Few

## Data Availability

All data are included in this paper. Additional information can be provided upon request to the correspondence author.

## Supplementary Materials

The following supporting information can be downloaded at: https://www.mdpi.com/article/10.3390/plants12040905/s1, Figure S1. Sampling design and field scene [25].
