# Supplementary material for: Effects of Tea Plant Varieties with High- and Low-Nutrient Efficiency on Nutrients in Degraded Soil
Source: Plants (Basel). 2023 Feb 16;12(4):905. doi: 10.3390/plants12040905 (PMC9959688; doi:10.3390/plants12040905)
Supplement: Supplementary file 1 [file plants-12-00905-s001.zip › plants-2119176-supplementary.pdf]

Supplementary Material

Effects of Tea Plant Varieties with High- and Low-Nutrient Efficiency on Nutrients in Degraded Soil

Li Ruan [1], Xin Li [2,3], Yuhang Song [2], Jianwu Li [1,2,*] and Kumuduni Niroshika Palansooriya [4]

[1] Institute of Sericulture and Tea, Zhejiang Academy of Agricultural Sciences, Hangzhou 310021, China
[2] Institute of Carbon Neutrality, Zhejiang A&F University, Hangzhou 311300, China
[3] Agricultural Technology Extension Station of Tangshan Agricultural and Rural Bureau, Tangshang 063000, China
[4] State Key Laboratory of Subtropical Silviculture, Zhejiang A&F University, Hangzhou 311300, China
[*] Correspondence: jameslee@zafu.edu.cn

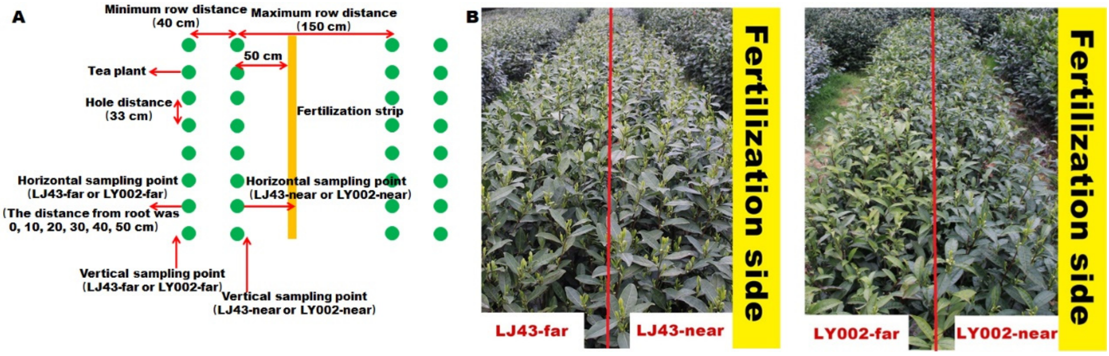

Figure S1. Sampling design and field scene. **A:** Sampling placements, planting and fertilization patterns; **B:** Tea growth phenotypes in fields [25].
